# Landscape configuration modulates the presence of leaf-cutting ants in eucalypt plantations

**DOI:** 10.1038/s41598-023-40426-9

**Published:** 2023-08-12

**Authors:** Pablo Cavigliasso, Ezequiel González, Abel Scherf, José Villacide

**Affiliations:** 1 INTA EEA Concordia, Estación Yuqueri s/n, Concordia, Entre Ríos Argentina; 2 Instituto Multidisciplinario de Biología Vegetal (IMBIV), CONICET - Universidad Nacional de Córdoba (UNC), Av. Vélez Sarsfield 1611, X5016GCA Córdoba, Argentina; 3 INTA EEA Montecarlo, Av. el Libertador 2472, Montecarlo, Misiones Argentina; 4 Grupo de Ecología de Poblaciones de Insectos, IFAB-INTA Bariloche, Modesta Victoria 4450, San Carlos de Bariloche, Rio Negro Argentina; 5 Present Address: INTA EEA Marcos Juárez, Ruta 12 km. 3, Marcos Juárez, Córdoba, Argentina

**Keywords:** Forestry, Ecosystem ecology, Biodiversity

## Abstract

Pest responses to landscape complexity show variable patterns globally, primarily related to species traits and specific managed habitats. Leaf-cutting ants (LCAs) are native insects and important pests of plantation forests in South America. We evaluated the responses of LCA nests in young *Eucalyptus* plantations to different spatial contexts: land uses, interfaces (adjacent land use pairs), agroecosystems, and landscapes. We selected 30 sites in the littoral region of Argentina representing three types of land uses neighboring *Eucalyptus* plantations: adult eucalypt plantations, citrus plantations, and semi-natural habitats. At each site, we quantified and identified LCA nests and characterized landscape composition and configuration in circles of 250 m radius. LCA nest abundance and presence were similar across different land uses, interfaces, and agroecosystems. Nest presence decreased in landscapes with increasing mean perimeter/area ratio and citrus coverage, whereas LCA abundance showed a similar trend. This indicates that heterogeneous landscapes and those with greater citrus plantation coverage were less likely to have LCA nests. Our findings suggest that landscape configuration was the main predictor of the LCA presence. Understanding the dynamics of LCAs populations and their complex associations with landscape components will contribute to developing successful environmental pest management strategies for plantation forests.

## Introduction

Intensification of agricultural production, including planted forests and fruit plantations, has significantly expanded in various regions over the last century, resulting in significant changes in landscape composition and a consequent simplification of its biological complexity^1,2^. Simplified productive landscapes are usually associated with higher pest pressure^3,4^ and lower levels of control by natural enemies^5^ in several crops. However, pest responses to landscape complexity show variable patterns globally, primarily related to species traits and the specific context in which crops are cultivated^6^. Furthermore, forestry systems are much less studied, and understanding how different key drivers modulate the spatiotemporal dynamics of pest species is crucial to developing effective management strategies, especially when dealing with native pest species^7^.

Plantation forests in tropical regions and in South America are typically developed as intensive crops of even-aged non-native tree species, cultivated in monoculture plots over large areas^7–9^. This regional pattern is likely due to the exceptional growth and yield rates observed in exotic trees, mainly eucalypts and pines, compared to those observed for the same species in their native distribution^10,11^. Additionally, these exotic trees exhibit higher productivity and shorter harvesting times than native tree species in the region. However, these non-native species have had only a short period of evolutionary adaptation to new insect-plant interactions, resulting in limited development of chemical and physical defenses against herbivory, particularly by native pest species^12–14^.

The distribution of generalist herbivore insect pest species is not limited to managed habitats such as forest plantation plots, but also extends to the surrounding areas, including borders and interfaces between different land uses^15^. These areas can have a significant impact on the population dynamics and behavior of pest species, as they provide a diverse range of resources and habitats. For example, habitat edges may have different microclimatic conditions, vegetation structure, and predator–prey interactions compared to the interior, which can affect the abundance and distribution of pest species^16^. Similarly, the interfaces between different land uses, such as agricultural fields and seminatural habitats, can create unique conditions that favor the establishment of pest species. At larger, landscape scales, the cover of semi-natural and managed habitats are relevant predictors of pest abundance, interacting with the type of cultivated habitat and the nativeness status of the pest species^6^. Therefore, native pests of exotic forest plantations need to be studied by incorporating different scales and habitats to better-understand how to manage them.

Leaf-cutting ants (LCAs) are a group of generalists’ herbivorous insects endemic to the Americas, from southern Argentina to the southern United States, except for Chile^12,17^. Grouped in the genera *Atta*, *Acromyrmex* and *Amoimyrmex*, all species harvest leaves and tender parts of the plants to nourish the gardens of mutualistic fungi (Agaricales: Basidiomycota) that they cultivate inside their nests^18^. LCAs are eusocial insects that exhibit a high level of social organization and some ecological and behavioral traits, such as overlapping generations and cooperative brood care, that may contribute to their ecological success and allow for rapid local adaptation^19–21^.

LCAs have become one of the main native pests for agricultural and forestry production from South America^12^. Despite that LCAs communities comprise several species, only a limited number of them generate impacts of economic importance for production (see examples in^17,22^). Currently, management actions for LCAs are typically focused on a pest- and crop-centric strategy^23^ and rely on the use of generic chemical insecticides applied within productive plots. Nevertheless, there is an urgent need for the forestry sector in particular, and society in general, to develop new management strategies for LCAs that are validated in productive environments and have a reduced environmental impact^7^. Therefore, for pest insect species such as LCAs that are highly polyphagous and their movement mechanisms allow them to exploit resources between different habitat patches, understanding how landscape characteristics influence dynamics is important to develop sustainable and environmentally safe actions within system-centric schemes^23^.

In our work, we evaluate how landscape components at different spatial scales modulate the LCA assemblage in young *Eucalyptus* planted forest in the Argentine Mesopotamia (Entre Ríos, Argentina). Specifically, we studied (a) the effect of the most representative land uses (LUs) present in the study region, which are young and mature eucalypt forest plantations, citrus plantations, and seminatural habitats, and (b) the effects of three types of interfaces, that is, areas where young *Eucalyptus* neighbor the other LUs, on presence and abundance of leaf-cutting ants. These interfaces were located in landscapes with varying heterogeneity and in two contrasting agroecosystems (forestation and mixed uses), allowing us to cover several spatial scales. The study area is one of the main regions of eucalypt production in Argentina, so improving our understanding of the spatial dynamics of LCAs populations is urgently needed. This information could be critical to generate management plans, plantation diagrams at extensive scales, and land-use planning associated with regions where these production systems are established or where progress is expected with their implementation.

## Results

**Figure 1:**
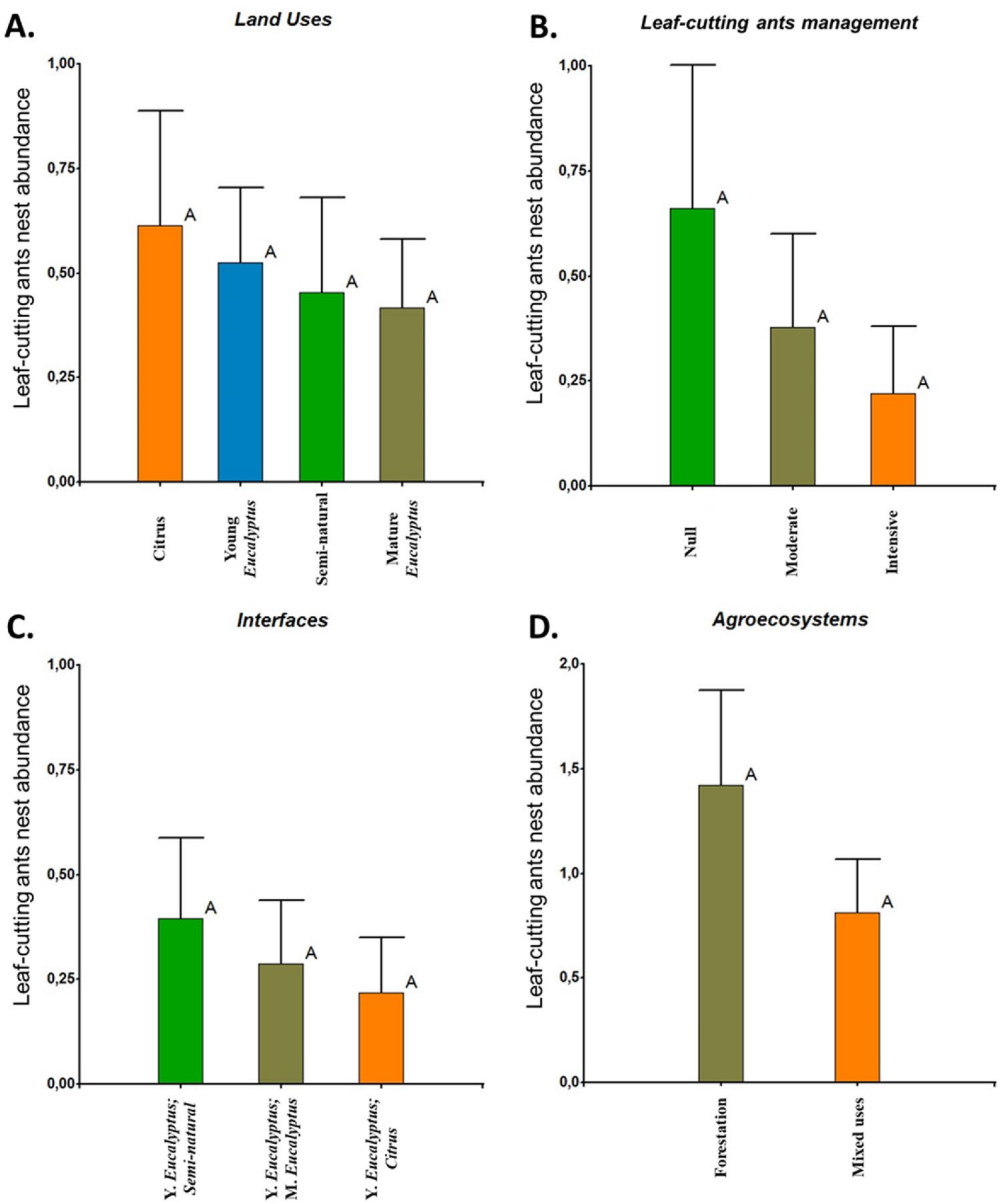
Comparative analyses between the categorical variables land uses (**A**), interfaces between LUs (**B**), LCA management associated with the LU contiguous to the Ey (**C**), and Agroecosystems where the different sites are included (**D**). The error bars represent standard errors.

A total of 36 LCA nests were detected, all of them belonging to the genus *Acromyrmex*. The community of LCAs was composed of two species of this genus. *Acromyrmex lundii* presented the highest abundance of nests (63.89%), followed by *Acromyrmex heyeri* (36.11%). When evaluating the relationships between the density of nests (abundance) and the categorical variables selected as descriptors of the studied region (LUs, Interfaces, Management associated with LCAs and Agroecosystems) we found no significant differences between the categories in all cases (GLMMs—Test-Wald: F_LU_ = 0.22, P_LU_ = 0.8840; F_Int_ = 0.41, P_Int_ = 0.6701; F_Man_ = 1.05, P_Man_ = 0.3650; F_Agr_ = 1.93, P_Agr_ = 0.1760) (Fig. 1A–D). Nevertheless, the Forest-Semi-natural interface was the one that concentrated the highest average (+ /− SE) abundance, with 1.27 (+ /− 0.45) nests of LCAs and the forestation agroecosystems presented 42.96% more nests of LCA than systems of mixed uses (1.42 + /− 0.46 and 0.81 + /− 0.25 nests, respectively). Similarly, LCA nest presence did not differ between LUs (F = 0.53, *p* = 0.4367), interfaces (F = 0.97, *p* = 0.3525), LCA management (F = 1.01, *p* = 0.2946), and agroecosystems (F = 0.06, *p* = 0.8126).

**Figure 2:**
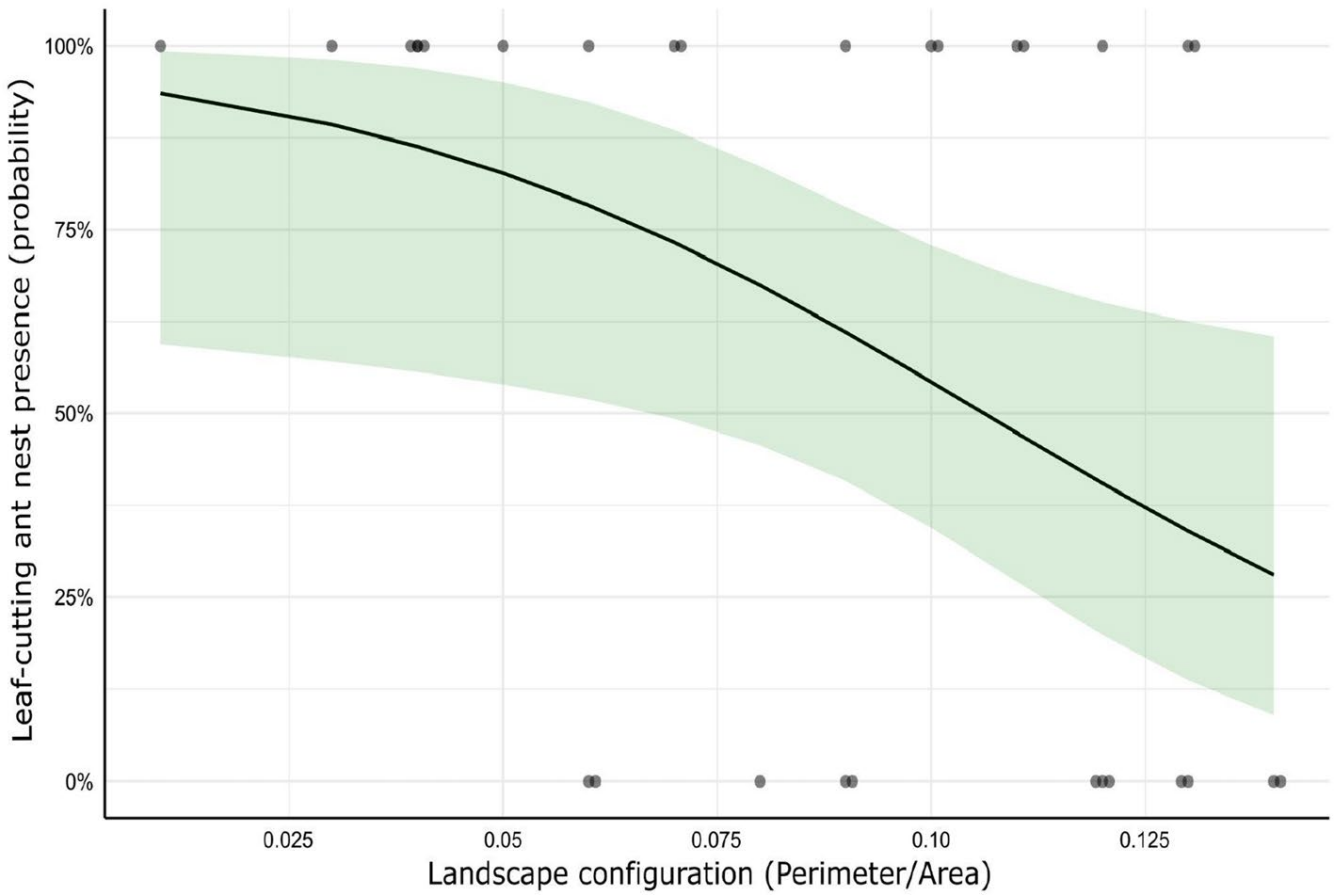
Landscape configuration (Perimeter/Area ratio) effects on LCA nest presence. The black line represents the effects predicted by the GLM and the green band the 95% confidence interval. Dots represent the presence/absence of nests at each of the study sites.

In general, neither the percentages of coverage of the dominant LUs nor the metrics of landscape configuration showed significant effects on the abundance of LCA nests. The best model included a marginally significant negative association with the Perimeter/Area ratio (*p* = 0.0584; Table S2; Fig. S1), whereas the two following competitive models included non-significant relationships with citrus cover and landscape heterogeneity. Similarly, the presence of LCA nests was negatively affected by the Perimeter/Area ratio (*p* = 0.0292; Table S2; Fig. 2), which denotes that more simplified productive landscapes with larger plots have a higher probability of hosting LCA nests. Most landscape composition metrics were not important for LCA nest presence, although the second-best model included a negative effect of citrus cover in the landscape (*p* = 0.0463; Table S2; Fig. S2).

## Discussion

In our study, we evaluated how leaf-cutting ant assemblages are modulated by several components that structure *Eucalyptus* plantations in the Argentine Mesopotamia at different spatial scales, from local (land uses, interfaces, LCA management intensities), to landscape (compositional and configurational heterogeneity) and regional scales (agroecosystems). Our findings suggest that the effect of individual land uses, and their interactions, are relatively weak, both locally and regionally. Whereas at the landscape scale, the configurational complexity of the system, estimated as perimeter/area of the patches, had the strongest effects on the presence /absence of the nests of LCA and a similar, though non-significant, trend on nest abundance.

Within the LCAs community of Entre Ríos, we found that *Acromyrmex* is the representative genus, with a prevalence of almost two-thirds of the species *A. lundii*. These results are consistent with recent surveys by Scherf et al.^24^ in systems with similar characteristics in the southeast of the Corrientes province (Argentina) in *Eucalyptus grandis* plantations. This species shared its presence in the registered community only with *A. heyeri*. Even so, we highlight that within the province of Entre Ríos, in addition to the reported species, six more species have been recorded within the genus *Acromyrmex*^25^ so we could, in a certain way, associate both *A. lundii* and *A. heyeri* to this type of productive agroecosystems.

Although no differences were found in the number of LCA nests between the studied land uses or in the different sampled interfaces, the interface constituted between young *Eucalyptus* plantations and semi-natural remnants was the one that seems to present the highest average density of LCA nests. This trend could reflect what has been reported in other works carried out within Argentina for this same genus^26,27^. In addition, studies carried out on changes in the nest density of the genus *Atta* in the Cerrado (Brazil), have shown similar patterns along a gradient of the different types of forests^28^. Similarly, we did not observe significant differences between the two regional agroecosystems (forestation vs. mixed uses), although the average values of LCA nests in the forestation tended to be 42.96% higher. Mixed-use systems have a higher representation of citrus plantations, where constant and intensive management of the LCA populations with chemically synthesized insecticides is performed^29^. Nevertheless, these regional differences in pest control strategies would only partly lead to lower abundance of LCA nests in these productive systems. In line with these results, we did not find statistical differences between the abundance of nests in focal *Eucalyptus* plantations in interface with land uses that have an intensive LCA management associated with the use of insecticides (citrus plantations) and those next to land uses with no management of LCAs (natural remnants). This shows that, on a local scale, conventional intensive management of LCA populations is not entirely efficient for their eradication^30^. Therefore, alternative approaches are needed to maintain a profitable and quality production while “coexisting” with these native organisms, which have a wide distribution and present complex strategies to associate with the surrounding environment.

Landscape composition (percentage of coverage of the dominant land uses and habitat diversity) does not seem to strongly influence the presence or abundance of LCA nests. Only an increase in the coverage of citrus plantations decreased the probability of finding LCA nests within the studied landscapes, which may be linked to the above-mentioned management in these habitats in a landscape scale. This pattern can be explained, at least in part, by the complex ecological behavior of LCAs, which includes the ability to use different resources for foraging and nesting found in the landscape^12^. Also, our results are consistent with the findings of Tamburini et al.^6^, which using a large global database found that native generalist pests did not respond to landscape composition gradients.

Few studies have contemplated how land use gradients affect the density of LCA nests per unit area. Ribero et al.^27^ used a similar approach to evaluate the effects of landscape components on LCA abundance and obtained similar results, with no differences in the abundance of LCA nests in mature and young eucalypt plantations It´s important to note that LCA nests are generally controlled with pesticides (such as, chlorpyrifos or sulfuramides) during the early stages of development of eucalypt plantations^10^, but not in the mature stage. Thus, other local regulation mechanisms, such as decreased understory vegetation cover, increased ground litter cover, and lower and more stable temperatures^11^, all of which are associated with mature plantations, can be linked with changes in LCA abundance and be more relevant than the cover of plantations. On the other hand, González et al.^31^ found that the richness and abundance of fungus-grower ants increased with native forest area and forest cover in the landscape, respectively. However, these native forest fragments were surrounded by soybean crops, where active control of LCAs is not performed.

The impact of landscape configuration on LCAs has not been previously explored in the literature. Our results show that eucalypt plantations inserted in simplified landscapes have a higher probability of presenting LCA nests than plantations found in landscapes with a higher mean perimeter/area ratio (smaller grain size, see^32^), while the same pattern, although not significant, was found for nest abundance. Different mechanisms related to habitat preference, spillover between land uses, and landscape-scale habitat availability could be linked to this effect. Edge habitats host pioneer plant communities that are preferred by LCAs and explain their higher abundance in forest edges^33–36^. In complex landscapes, edge habitats are much more available, and the resources preferred by ants may be diluted, as observed for other pests^37,38^, whereas in simple landscapes ants would tend to accumulate in the few available edges. On the other hand, edges are linked to spillover between habitats, which is one of the most important moderators of organisms and communities at the landscape scale^39^, including managed and natural ecosystems^46^. Spillover between habitats often increases with increasing edge density (i.e., perimeter-to-area ratios), and this landscape complexity can benefit organisms by facilitating resource use in different habitats^40^. At the same time, more complex landscapes have been linked to greater plant and habitat diversity^41–44^, which would translate into more resources for generalist herbivores such as ants and consequently decrease the attractiveness of young *Eucalyptus* plantations.

## Concluding remarks

We found that the configuration of the landscape, characterized by the perimeter/area of the different patches, was the main predictor of the presence of LCAs, which could be linked to a resource dilution in complex landscapes, where young *Eucalyptus* forests have lower pest pressure. In contrast, local characteristics in or near a forest plantation, that is characterized by a high homogenization of its spatial context and its associated resources, were not so relevant. Therefore, decreasing patch sizes within productive landscapes by planting small plots of trees surrounded by other habitats could be the simplest way to decrease damage by LCAs. On the other hand, we can infer that LCAs are likely highly resistant to human disturbance, as this group of ants is well represented along a gradient of different habitats with contrasting management histories^45^. Based on all the available information, we suggest that the dynamics of LCA communities could be modulated at scales larger than the forest plot where management is usually performed. Thus, future studies should attempt to address these questions at scales such as landscapes or ecoregions. This information would be very valuable for understanding the dynamics of these ant populations and their complex association with landscape components, the basis of a successful integrated management plan for *Eucalyptus* plantations.

## Methodology

### Study area

The study was carried out in a region that belongs to the Phytogeographic province of Espinal^46^ and is characterized by fruit and forestry production and urban development, which are linked to marked reductions of native vegetation for decades^47^. Due to the climate and soils of this region located in the Uruguay River basin (31°47′38.67 S; 58°18′51.04″ W), large-scale plantations of *Eucalyptus sp. afin grandis* (> 500 ha) are predominant. The planting density is usually between 600 and 900 trees/ha that are harvested at 10–15 years, resulting in dense and uniform monocultures with little understory vegetation and closed canopies. In the department of Concordia (31°23′29.21 ″ S; 58°01′02.33″ W) conventional citrus fruit plantations (plots < 50 ha) are predominant, although blueberries, pecans and small blocks of *Eucalyptus sp.* (< 20 ha) are common, creating a mosaic of different land uses.

Throughout the entire argentine Mesopotamia, a marked conversion is taking place at the landscape level due to the increase of forest plantations for the production of wood, cellulose and biomass^48–51^. This is fostered by favorable policies that promote forest planting under Laws No. 25,080 and No. 26,432. Currently, in Argentina there are more than 1.3 million hectares of forest plantations, providing more than 90% of the wood used in our country^52^. In the last decade, a significant reduction (~ 50%) of the surface covered by representative forests of Espinal has been registered in the region, and in 12% of the cases the tendency is to replace the Espinal by crops and forest plantations (information from PIRE project, NSF grant n° 124,344).

### Sampling design

**Figure 3:**
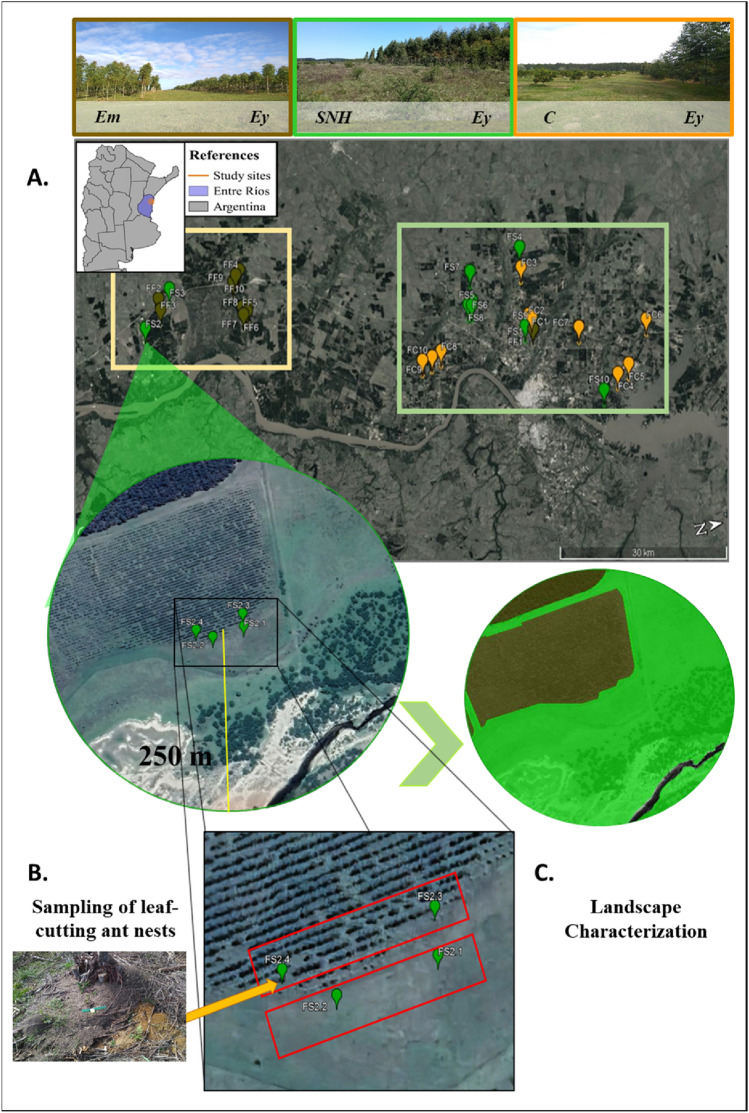
Experimental design diagram. (**A**) Location of the 30 sites in space. As an example, the images above show the three interfaces under study. Boxes of different colors on the map highlight contrasting agroecosystems (yellow: forestation; light green: mixed uses). (**B**) The bottom left image shows the location of the sampling areas. The rectangular polygons (red) represent the band transects and the points the position of the LCA nests. (**C**) The bottom right image represents the process of characterizing LUs within the landscape. Different colors denote different land uses. **References: Ey**) young Eucalyptus plantations (~ 3–4 years); **Em**) mature Eucalyptus plantations (8–15 years); (**C**) Citrus plantations; **SNH**) Semi-natural remnants. Satellite images were obtained to free use from QGIS servers, assembled using the Print Composer tool, and processed using the Google Satellite option of the OpenLayers plugin of QGIS “Białowieża” 3.22.5 (77).

We surveyed LCA communities in 30 sites selected based on their landscape components (see details for each site in Table S1 and the experimental design diagram in Fig. 3). In all cases, *young Eucalyptus plantations* (~ 3–4 years) (**Ey**) were our focal landscape components due to the major problems caused by LCAs defoliation. Three different land-use (LU) combinations that represent different interfaces with the Ey were selected: *mature Eucalyptus plantations*(8–15 years) (**Em**, n = 10), *Citrus plantations* (**C**, n = 10) and *Semi-natural remnants* (i.e., Espinal; **SNH**, n = 10). Each of the LU combinations with the Ey represented differential LCA management intensities that would, initially, affect the potential sources of LCA nests (*Intensive*, **Ey:C**, constant management of LCAs using insecticides; *Moderate*, **Ey:Em**, management only during the first years of the plantation with insecticides; *Null*, **Ey:SNH**, LCAs are not handled with insecticides). These categories were used as one of our predictor variables in the analysis where, unlike the interface category, only considers the nests present in the LU adjacent to the Ey and not those located within it. In addition, the studied sites were inserted in two different agroecosystems: *Forestation* (n = 11), an area with a predominance of forest production and practically no fruit growing; *Mixed Uses* (n = 19), an area with a predominance of fruit production and with small islands of forest plantations (~ 20 ha).

### Sampling of leaf-cutting ants

LCA communities at each site were surveyed visually during April–June 2022 using band transects (50 * 6 m) located parallel to the edge of each LU (E_y_:Em, C, SNH). Each band transect contained 3 m of the inner and outer edge of the LUs that defined the interfaces. This sampling location was chosen because previous studies reported a low or null presence of LCA nests in the central area of the plantation forest patches^53–55^. Therefore, each site is represented by the information of 2 transects (sampled area/site: 600 m^2^). Each transect was covered systematically, going back and forth, observing strips of 3 m each time. For each LCA nest, the geographical position of the nest was recorded, and samples were taken from individuals that were on the foraging routes for their taxonomic identification. Given that LCAs tend to move their nests after rain, sampling was carried out at least 2 days after rainy days. Ant samples collected in each nest were classified at the species level under a stereoscopic magnifying glass using dichotomous keys^56–59^, reference collections, and with the help of specialized taxonomists.

### Landscape characterization

To understand the role of landscape heterogeneity, we classified LUs within a circular polygon (250 m radius) at each of the 30 selected sites. This scale was selected based on previous studies that already detected landscape effects on LCAs and other ant groups using this radius^31,60^. Moreover, ants foraging distances away from their nest are usually much smaller^61^, and our main interest was linked to the potential foraging on young eucalypt plantations. The geographic location of the centroid of each site was located in the intermediate zone between each pair of transects, at 25 m from their ends.

To evaluate the influence of the compositional heterogeneity of the landscape, four representative LUs were identified: *Citrus*: area occupied by citrus plantations under conventional productive management; *Young Eucalyptus*: Implanted blocks, mainly of *E. grandis*, under conventional management associated with the first 4 years of growth; *Adult Eucalyptus*: Implanted blocks, mainly of *E. grandis*, under conventional management associated with mature plantations; *Semi-natural*: areas that include natural reserves, abandoned lots, recovery areas, roadsides, and lowlands, among others. Other land uses (e.g., urban developments) were not considered in the analysis due to their low representation, but their cover was quantified during landscape characterization.

To assess the influence of the number of different land uses and their representation within the landscape, the area and perimeter of each patch of the LUs were used to calculate the “habitat diversity” index (exp H′, where H′ is the Shannon diversity index-Wiener^62^) and the average “Perimeter/Area” ratio of all patches at each site as measures of landscape complexity within each circle (composition and configuration, respectively). We used the “Google Satellite” option of the “OpenLayers plugin” tool of QGIS “Białowieża” 3.22.5^63^, with a WGS/Pseudo Mercator projection (EPSG: 3857) for the calculation of spatial metrics from satellite imagery from May 2022.

### Statistical analysis

We modeled the presence and abundance of LCA nests (response variables) per transect using generalized linear mixed models (GLMMs;^64^). LCA abundance was modeled using a Poisson error distribution, whereas for LCA presence we used a binomial error distribution. The variables “Interface”, “Management” and “Agroecosystem” were used as fixed factors in separate models. Spatial replicates (“Site”) and transects corresponding to “LUs” describing each “Interface” were added as random factors, with land uses nested within each site. In addition, the “LUs” factor was evaluated independently, comparing the abundance and presence of LCA per transect between each category. In this last case we use only “Site” as a random variable.

To evaluate the effects of compositional and configurational heterogeneity of the landscape on LCA nest abundance and presence, we used GLMs with presence and total abundance per site (sum of the two transects) as the response variables. The proportions of the different LU cover (compositional heterogeneity), the “habitat diversity” index and the “Perimeter/Area” ratio (configurational heterogeneity) were used as fixed factors. All analyses were performed using the software R (version 4.1.2;^65^). The background hypotheses proposed in this study are presented below (Table 1). The comparisons between LUs, Interfaces, Managements and Agroecosystems were analyzed in simple models and reported using Fisher’s Least Significant Difference (LSD) tests. For landscape analyses, we fitted separate models with each landscape metric and used the Akaike Information Criterion for small sample sizes (AICc) to select the model that with the lowest AIC value and competitive models within a ΔAICc = 2^66^. We used the *glm, glmer* and *glmer.nb* functions of the “lme4” package version 1.1–27.1 to fit the models^67^ and the *ggpredict* function of the “ggeffects” package version 1.1.1^68^ and the graphic tools of the “ggplot2” package version 3.3.5 for visual representations^69^. For all models, the residuals were checked using the package DHARMa^70^ and no issues were detected. Furthermore, using the same package we tested for spatial autocorrelation using Moran’s I tests for distance-based autocorrelation, which was not significant for any of the models.

**Table 1:** Background hypothesis and candidate models for the density and presence of LCA (x).

Background hypothesis	Proposed model
Null model	x ~ 1
Effects of LUs	x ~ LUs type (Ey, Em, C, SNH)
Effects of interface between LUs	x ~ Interface type (Ey:Em, Ey:C; Ey:SNH)
Effects of management of LCAs associated with the LU adjacent to the Ey	x ~ Management of leaf-cutting ants (Ey_Em_, Ey_C_, Ey_SNH_)
Effects of the agroecosystem where the studied sites are inserted	x ~ Agroecosystem (Mixed uses vs. Forestation)
Effects of landscape structure	x ~ LUs cover (%); PE/AR; exp H′

### Supplementary Information

Supplementary Information.

## Data Availability

All the raw data used for the analyses are provided in Table S1 as Supplementary Information.
