# Supplementary material for: Landscape configuration modulates the presence of leaf-cutting ants in eucalypt plantations
Source: Sci Rep. 2023 Aug 12;13:13130. doi: 10.1038/s41598-023-40426-9 (PMC10423229; doi:10.1038/s41598-023-40426-9)
Supplement: Supplementary file 1 — Supplementary Information. [file 41598_2023_40426_MOESM1_ESM.docx]

SUPPLEMENTARY INFORMATION

**Landscape configuration modulates the presence of leaf-cutting ants in eucalypt plantations**

Cavigliasso, Pablo; González, Ezequiel; Scherf, Abel & Villacide, José

| **Table S1-** Detailed information of each studied site (n=30), its landscape structure included within the circular polygons and the LCA metrics used as response variables for the analyses. | | | | | | | | | | | | | | | | | | | | |
| --- | --- | --- | --- | --- | --- | --- | --- | --- | --- | --- | --- | --- | --- | --- | --- | --- | --- | --- | --- | --- |
|  | |  | |  | **Geographical coordinates ^a^** | | | **Coverage (%) ^b^** | | | | | | **Configuration** | | |  | **LCAs nest density** | | |
| **Agroecosystem** | **Interface** | | **Site** | | | **Lat.** | **Long.** | | **C** | **Fm** | **Fy** | **Forestation**  **(Total)** | **SNH** | | **HD ^c^** | **PE/AR^d^** | **Presence^e^** | | **Fy** | **LU _Interface_** |
| Forestation | FyFm | | FF10 | | | -31.3624821 | -58.1202742 | | 0.00 | 46.85 | 28.51 | 75.37 | 16.94 | | 3.49 | 0.12 | 1 | | 0 | 3 |
|  | FyFm | | FF2 | | | -31.3655749 | -58.102671 | | 0.00 | 0.00 | 79.57 | 79.57 | 18.79 | | 2.60 | 0.08 | 0 | | 0 | 0 |
|  | FyFm | | FF3 | | | -31.3656228 | -58.1025817 | | 0.00 | 17.73 | 54.44 | 72.16 | 25.94 | | 3.49 | 0.09 | 0 | | 0 | 0 |
|  | FyFm | | FF4 | | | -31.3657112 | -58.2230562 | | 0.00 | 43.10 | 12.57 | 55.68 | 30.97 | | 3.58 | 0.06 | 1 | | 1 | 0 |
|  | FyFm | | FF5 | | | -31.4488981 | -58.2198501 | | 0.00 | 23.19 | 49.92 | 73.11 | 23.32 | | 3.55 | 0.06 | 0 | | 0 | 0 |
|  | FyFm | | FF6 | | | -31.455021 | -58.1723618 | | 0.00 | 29.74 | 60.77 | 90.50 | 9.42 | | 3.18 | 0.05 | 1 | | 2 | 0 |
|  | FyFm | | FF7 | | | -31.4574279 | -58.1652402 | | 0.00 | 48.38 | 40.46 | 88.83 | 10.84 | | 3.31 | 0.10 | 1 | | 0 | 2 |
|  | FyFm | | FF8 | | | -31.5354378 | -58.0978789 | | 0.00 | 35.92 | 48.48 | 84.40 | 5.72 | | 3.14 | 0.10 | 1 | | 1 | 0 |
|  | FyFm | | FF9 | | | -31.5520506 | -58.0832515 | | 0.00 | 70.89 | 19.47 | 90.35 | 9.28 | | 3.01 | 0.04 | 1 | | 0 | 1 |
|  | FySNH | | FS2 | | | -31.987206 | -58.252939 | | 0.00 | 4.37 | 26.79 | 31.15 | 67.12 | | 3.08 | 0.11 | 1 | | 2 | 2 |
|  | FySNH | | FS3 | | | -31.8277192 | -58.3060962 | | 0.00 | 4.70 | 34.75 | 39.46 | 55.01 | | 3.30 | 0.07 | 1 | | 4 | 0 |
| Mixed uses | FyC | | FC1 | | | -31.2693159 | -57.9861721 | | 12.63 | 34.23 | 13.75 | 47.98 | 38.36 | | 4.48 | 0.14 | 0 | | 0 | 0 |
|  | FyC | | FC10 | | | -31.551872 | -58.093811 | | 46.75 | 30.50 | 0.00 | 38.54 | 13.22 | | 3.68 | 0.14 | 0 | | 0 | 0 |
|  | FyC | | FC2 | | | -31.2747326 | -57.9796102 | | 32.99 | 30.28 | 4.78 | 38.19 | 28.66 | | 4.41 | 0.13 | 0 | | 0 | 0 |
|  | FyC | | FC3 | | | -31.366883 | -58.209275 | | 46.14 | 17.33 | 14.11 | 37.95 | 11.77 | | 4.32 | 0.12 | 0 | | 0 | 0 |
|  | FyC | | FC4 | | | -31.242611 | -58.998856 | | 52.06 | 32.57 | 0.00 | 35.93 | 12.01 | | 3.61 | 0.12 | 0 | | 0 | 0 |
|  | FyC | | FC5 | | | -31.222311 | -58.010433 | | 16.95 | 57.77 | 0.00 | 60.68 | 20.02 | | 3.46 | 0.13 | 1 | | 2 | 0 |
|  | FyC | | FC6 | | | -31.180886 | -58.076206 | | 32.20 | 5.68 | 45.45 | 51.14 | 16.53 | | 4.15 | 0.04 | 1 | | 0 | 4 |
|  | FyC | | FC7 | | | -31.291519 | -58.089089 | | 8.09 | 64.84 | 0.00 | 64.84 | 24.24 | | 3.21 | 0.04 | 1 | | 0 | 3 |
|  | FyC | | FC8 | | | -31.519319 | -58.102211 | | 44.67 | 26.02 | 3.34 | 32.61 | 19.73 | | 4.17 | 0.13 | 0 | | 0 | 0 |
|  | FyC | | FC9 | | | -31.535433 | -58.096175 | | 36.46 | 23.16 | 6.80 | 31.49 | 30.22 | | 4.52 | 0.13 | 1 | | 1 | 0 |
|  | FyFm | | FF1 | | | -31.3591744 | -58.1172964 | | 0.00 | 40.74 | 6.03 | 46.78 | 40.24 | | 3.33 | 0.09 | 1 | | 1 | 0 |
|  | FySNH | | FS1 | | | -31.8258046 | -58.2376123 | | 12.96 | 14.02 | 8.96 | 22.98 | 61.61 | | 3.86 | 0.11 | 1 | | 0 | 2 |
|  | FySNH | | FS10 | | | -31.269361 | -57.977042 | | 0.00 | 0.00 | 35.02 | 35.02 | 64.29 | | 2.86 | 0.01 | 1 | | 0 | 2 |
|  | FySNH | | FS4 | | | -31.9380831 | -58.3090634 | | 0.00 | 0.34 | 34.47 | 34.81 | 63.51 | | 2.91 | 0.12 | 0 | | 0 | 0 |
|  | FySNH | | FS5 | | | -31.9380842 | -58.3092252 | | 0.00 | 37.85 | 38.60 | 76.45 | 23.00 | | 3.64 | 0.03 | 1 | | 1 | 0 |
|  | FySNH | | FS6 | | | -31.9382588 | -58.3093725 | | 0.00 | 1.31 | 35.56 | 36.86 | 61.14 | | 3.03 | 0.04 | 1 | | 1 | 0 |
|  | FySNH | | FS7 | | | -31.9582074 | -58.2968455 | | 0.00 | 34.08 | 22.99 | 57.07 | 40.13 | | 3.80 | 0.06 | 0 | | 0 | 0 |
|  | FySNH | | FS8 | | | -31.9870769 | -58.2527259 | | 0.00 | 27.20 | 20.70 | 47.90 | 49.35 | | 3.76 | 0.09 | 0 | | 0 | 0 |
|  | FySNH | | FS9 | | | -31.377183 | -58.111142 | | 0.00 | 24.26 | 11.72 | 35.98 | 63.48 | | 3.38 | 0.07 | 1 | | 1 | 0 |

1. geographic coordinates in decimal degrees; (b) percent of vegetation cover within a circular polygon of 250 m radius; (c) habitat diversity index; (d) Perimeter/Area ratio; (e) 1: nest presence, 0: nest absence**.** See more details in the *Landscape characterization* section of the Methodology

**Table S2 -** Best models for the influence of landscape composition and configuration on LCAs nest presence and abundance in Entre Ríos, Argentina. For each response variable, the AICc, ΔAICc, estimates (± SE) are shown for all models. Significant effects are highlighted in bold.

| **Response variable** | **Predictor variable** | **AIC** | **ΔAICc** | **Slope estimate (± SE)** |
| --- | --- | --- | --- | --- |
| LCA nest presence | **Perimeter/Area** | **38.87** | **0.00** | **-27.86 (12.77)** |
|  | **Citrus cover** | **40.16** | **1.29** | **-0.05 (0.02)** |
|  | Landscape heterogeneity | 41.82 | 2.95 | -1.37 (0.84) |
|  | Old forest cover | 43.63 | 4.76 | 0.02 (0.02) |
|  | Total forest cover | 43.98 | 5.11 | 0.02 (0.02) |
|  | Natural habitat cover | 44.41 | 5.54 | 0.01 (0.02) |
|  | Young forest cover | 44.82 | 5.95 | 0.001 (0.02) |
| LCA nest abundance | Perimeter/Area | 92.90 | 0.00 | -8.41 (4.45) |
|  | Citrus cover | 93.64 | 0.74 | -0.02 (0.01) |
|  | Landscape heterogeneity | 94.17 | 1.27 | -0.53 (0.36) |
|  | Natural habitat cover | 95.52 | 2.62 | 0.01 (0.01) |
|  | Young forest cover | 96.17 | 3.27 | 0.004 (0.01) |
|  | Old forest cover | 96.43 | 3.53 | -0.002 (0.01) |
|  | Total forest cover | 96.48 | 3.58 | 0.001 (0.01) |


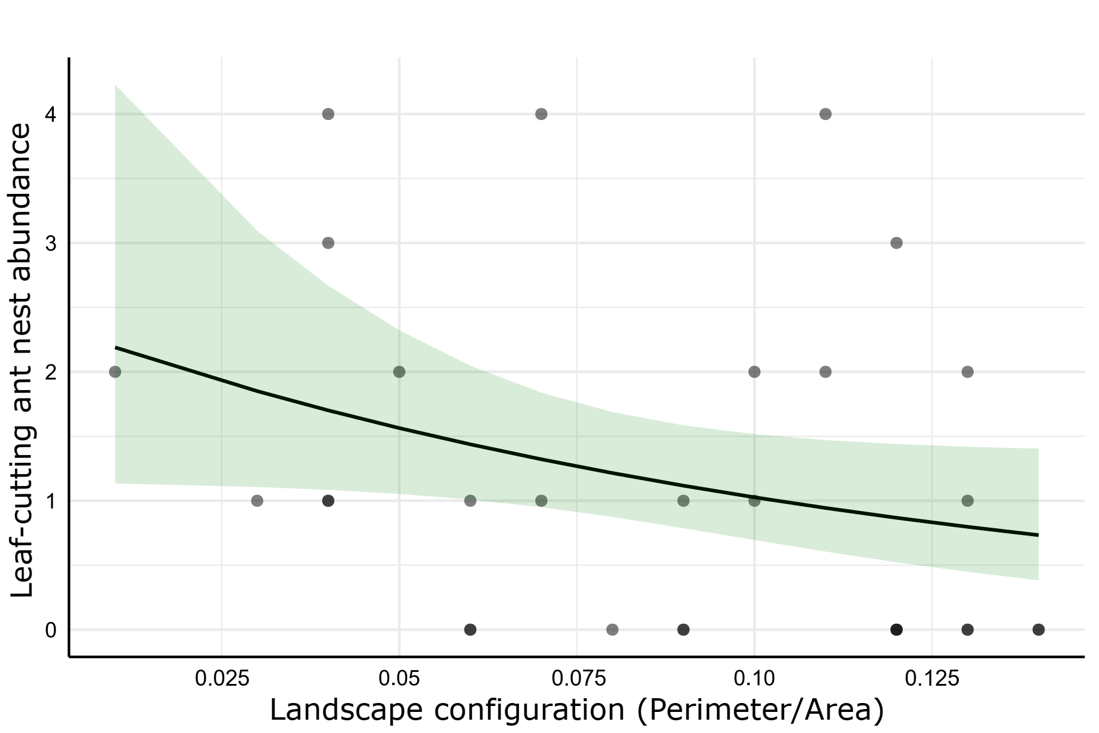


**Figure S1.** Landscape configuration (Perimeter/Area ratio) effects on LCA nest abundance. The black line represents the effects predicted by the GLM and the green band the 95% confidence interval. Dots represent the abundance of nests at each of the study sites.


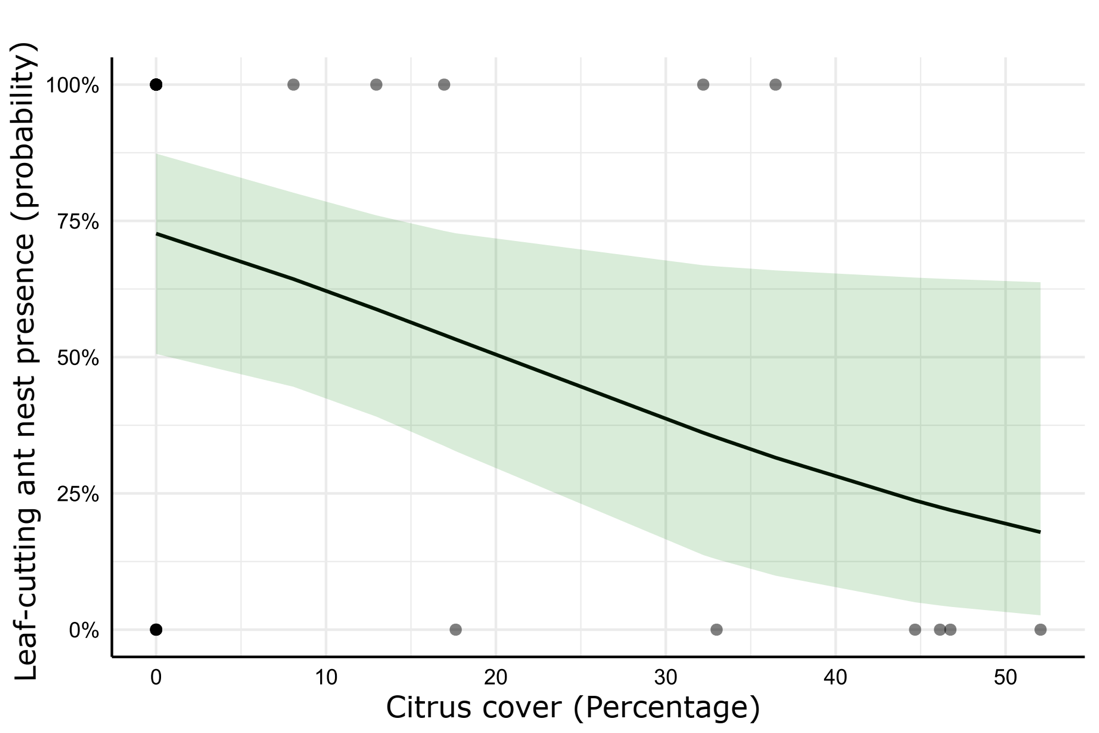


**Figure S2.** Citrus cover in the landscape effects on LCA nest presence. The black line represents the effects predicted by the GLM and the green band the 95% confidence interval. Dots represent the presence/absence of nests at each of the study sites.
